# The Value of Clinical Frailty Scale (CFS) as a Prognostic Tool in Predicting Mortality in COVID-19—A Retrospective Cohort Study

**DOI:** 10.3390/ijerph19031104

**Published:** 2022-01-19

**Authors:** Magdalena Jachymek, Aleksandra Cader, Michał Ptak, Wojciech Witkiewicz, Adam Grzegorz Szymański, Katarzyna Kotfis, Jarosław Kaźmierczak, Aleksandra Szylińska

**Affiliations:** 1 Department of Cardiology, Pomeranian Medical University, Powstańców Wielkopolskich 72, 70-111 Szczecin, Poland; magdajachymek@gmail.com (M.J.); witkiewiczwojciech@gmail.com (W.W.); adam.szymanski4666@gmail.com (A.G.S.); jar.kazmierczak@o2.pl (J.K.); 2 Department of Nephrology, Transplantology and Internal Medicine, Pomeranian Medical University, Powskańców Wielkopolskich 72, 70-111 Szczecin, Poland; aleksandra.cader@wp.pl (A.C.); michalptak@mail.com (M.P.); 3 Department Anesthesiology, Intensive Therapy and Acute Intoxications, Pomeranian Medical University, Powstańców Wielkopolskich 72, 70-111 Szczecin, Poland; katarzyna.kotfis@pum.edu.pl; 4 Department of Medical Rehabilitation and Clinical Physiotherapy, Pomeranian Medical University in Szczecin, Żołnierska 54, 71-210 Szczecin, Poland

**Keywords:** clinical frailty scale, COVID-19, SARS-CoV-2, coronavirus, outcome, mortality

## Abstract

Background: Due to the unpredictable nature of COVID-19, there is a need to identify patients at high risk of severe course of the disease and a higher mortality rate. Objective: This study aims to find the correlation between frailty and mortality in adult, hospitalized patients with COVID-19. Methods: Clinical records of 201 patients who suffered from COVID-19 and were hospitalized between October 2020 and February 2021 were retrospectively analyzed. Demographic, clinical, and biochemical data were collected. Patients were assessed using Clinical Frailty Scale (CFS) and were divided into three groups: CFS 1–3 fit; CFS 4–6 vulnerable and with mild to moderate frailty; CSF 7–9, severe frailty. The association between frailty and in-hospital mortality was the primary outcome. Results: Severe frailty or terminal illness was observed in 26 patients (12.94%) from a cohort of 201 patients. Those patients were older (median age 80.73, *p* < 0.001) and had more comorbidities. Frailty was also associated with higher requirement for oxygen supplementation, greater risk of in-hospital complications and worse biochemical laboratory results. An increase in CFS score also correlated with higher mortality (OR = 1.89, *p* < 0.001). The Conclusions: Clinical Frailty Scale (CFS) can be used as a potentially useful tool in predicting mortality in patients with COVID-19.

## 1. Introduction

The coronavirus Disease 2019 (COVID-19) pandemic put the global healthcare system to the test. Many countries, including Poland, were unable to provide patients with adequate care due to the insufficient number of hospital beds for patients requiring isolation during treatment of COVID-19. This situation pushed governments to create places such as temporary hospitals, mainly by rearranging existing wards but also by creating new hospitals. The latter was the case with a Temporary Hospital in Szczecin, Poland. 

After two years of the pandemic, we already know that most COVID-19 cases are mild or asymptomatic, and many patients do not require hospital care. From the group of patients presenting with mild or moderate symptoms it was essential to identify those with a higher risk of rapid deterioration of respiratory function and probability of death and therefore who might require hospitalization and more intensive treatment. Being at the front line of the COVID-19 war, we wanted to find a simple tool to quickly identify patients with a poor clinical prognosis. 

Numerous studies have suggested different risk factors of increased mortality and necessity for invasive ventilation in COVID-19 such as: older age, male sex, comorbidities or multimorbidity, high respiratory rate, radiographic severity score, elevated neutrophile count, higher C-reactive protein and creatinine concentration, low albumin, and blood oxygen saturation below 93% [1,2].

Frailty is a state of reduced ability to recover from a stressful event, which appears with older age, and is associated with increased risk of adverse outcomes and death. One of the most popular tools to grade frailty is the Clinical Frailty Scale (CFS), originally created in 2005 as a 7-point scale [3] and later modified and expanded to a 9-point scale [4]. CFS positions a patient’s frailty from very fit (CFS 1) to terminally ill (CFS 9), i.e., someone whose life expectancy is below six months.

The National Institute for Health and Care Excellence (NICE) provided guidelines, which recommend that patients admitted to hospital with COVID-19 should be assessed with the Clinical Frailty Scale (CFS) to evaluate baseline health and to consider treatment expectations [5]. According to these guidelines, all patients admitted to the Temporary COVID-19 Hospital in Szczecin were assessed with CFS to predict health outcomes. Based on gathered information, our study aimed to confirm a high the CFS score as an independent risk factor of death for patients suffering from COVID-19, who required hospital treatment. Our secondary purpose was to analyze treatment strategies and possible complications according to CFS score.

## 2. Material and Methods

We conducted an observational retrospective cohort study. The data come from three ad-hoc high-dependency acute care units (“oxygen units”) from two temporary hospitals for SARS-COV 2 infected patients in Szczecin, Poland, hospitalized between October 2020 and February 2021. Available oxygen therapies included: passive oxygen flow, high-flow nasal oxygen therapy (HFNOT), and non-invasive ventilation (NIV). Patients requiring invasive ventilation were transferred to another unit (“ventilation unit” as an equivalent to an ICU). 

### 2.1. Ethical Considerations

The study received a waiver from the Bioethical Committee of the Pomeranian Medical University because of its retrospective and observational nature (decision no. KB-0012/15/02/2021/Z dated 3 February 2021).

### 2.2. Study Population

The inclusion criteria were age over 18 years old and confirmed SARS-COV 2 infection with either reverse transcription polymerase chain reaction (RT-PCR) or antigen test. We excluded terminally ill patients without active COVID-19, who died because of other severe diseases soon after admission.

### 2.3. Data Pollection

Data were retrieved from an electronic hospital database and included: study group characteristics (demographic data, comorbidities, addictions), medications, symptoms, laboratory results and respiratory parameters on admission, applied treatment, complications, and follow-up. The Charlson Comorbidity Index (CCI) was used and calculated using and on-line open-source calculator. Some results from analysis of this database regarding delirium in COVID-19 have already been published [6]. Patients were assessed using the CFS by medical doctors on admission, none of whom were geriatricians, trained according to the recent guidelines [7]. We divided patients into three groups according to CFS score: CFS 1–3, patients without frailty; CFS 4–6, patients vulnerable and with mild to moderate frailty, CFS 7–9, patients with severe frailty to terminally ill, similarly to previous authors [8].

### 2.4. Statistical Analysis

All analyses were performed using licensed software Statistica 13 (StatSoft, Inc., Tulsa, OK, USA). The continuous variables are presented as mean, standard deviation (SD), and median. The categorical variables are presented as numbers and a percentage. For statistical significance, we used multiple comparison test. Chi-square test and Fisher test were used to compare qualitative data. We performed a receiver operating characteristic (ROC) analysis to evaluate which parameters have the major impact on mortality. We report AUC (with 95% CI) for age, continuous CCI, CFS, and PaO_2_/FiO_2_. Kaplan-Meier analysis calculated the probability of survival. The relationship between the analyzed parameters was evaluated using logistic regression model analysis. The multivariable logistic regression was corrected for potentially distorting data age, gender, BMI, and comorbidities. Statistical significance was set at *p*-value ≤ 0.05.

## 3. Results

A total of 201 patients were included in the study: 129 patients (64.18%) without frailty (CFS 1–3), 46 patients (22.89%) with mild or moderate frailty (CFS 4–6) and 26 patients (12.94%) with severe frailty to terminally ill (CFS 7–9). 

Table 1 presents demographic data, addictions, and co-morbidities. Patients from higher CFS group were older (*p* < 0.001) and had more comorbidities (hypertension (*p* < 0.001), chronic heart failure (*p* < 0.001), atrial fibrillation (*p* < 0.001), previous ischemic stroke (*p* < 0.001), internal carotid artery stenosis (*p* = 0.004), chronic peripheral ischemia (*p* = 0.003), previous venous thrombosis (*p* < 0.001) than patients with low CFS score. We decided to calculate the Charlson Comorbidity Index (CCI) to sum up comorbidities and predict 10-year survival. Patients with higher CFS also had higher CCI (*p* < 0.001). Diabetes treated with insulin (*p* < 0.001), chronic kidney diseases (CKD) (*p* = 0.004) and chronic coronary syndrome (CCS) (*p* < 0.001) with myocardial infarction (MI) (*p* = 0.005) were most frequent in the CFS 4–6 group. Patients without frailty (CFS 1–3) most frequently smoked cigarettes (*p* = 0.011).

Medications taken by the patients before admission are shown in Table 2. Patients with CFS (7–9) more often were treated with acetylsalicylic acid (ASA) (*p* < 0.016), new oral anticoagulants/oral anticoagulants (NOAC/OAC) (*p* = 0.003), calcium channel blockers (CCBs) (*p* = 0.049), statins/fibrates (*p* = 0.006), nitrates (*p* = 0.016), diuretics (*p* = 0.022), opioids (*p* = 0.024). Insulin (*p* < 0.001) and immunosuppression (*p* = 0.025) were more widespread in the CFS 4–6 group. Bronchodilators were taken most often by patients without frailty (*p* = 0.05), less often in the mild to moderate frailty group and finally these drugs were not used before admission by the frailest patients.

Table 3 shows coronavirus-related symptoms on admission to the hospital. Reported symptoms differed among groups. Patients with the highest CFS more often suffered from nausea (*p* = 0.003) and vomiting (*p* = 0.010). Interestingly, these symptoms were not registered in the moderate frailty group. Patients with CFS 1–3 more often complained of cough (*p* = 0.011), lack of taste (*p* < 0.001), and lack of smell (*p* = 0.042).

Laboratory results on admission are shown in Table 4. Most results were worse in the severe frailty group (CFS 7–9). Only INR, CKMB and TnT were the highest in the mild to moderate frailty group (CFS 4–6). APRI (AST to platelet ratio index - determining liver function) was equally high in CFS 1–3 and 7–9 groups.

As presented in Table 5, there were significant differences in respiratory parameters and type of oxygen therapy applied on admission between groups. Patients with CFS 4–6 and CFS 7–9 required more intense oxygen supplementation with non-rebreather masks (*p* = 0.009) and HFNOT or NIV (*p* = 0.018) with higher oxygen flows (*p* = 0.009), and fraction of inspired oxygen (FiO_2_) in the inhaled mixture of gases (*p* = 0.006). Furthermore, the oxygenation ratio (partial pressure of oxygen/fraction of inspired oxygen (PaO_2_/FiO_2_) was higher in more frail patients (*p* = 0.017). Acute respiratory distress syndrome (ARDS) occurred predominantly in patients without frailty, but the severity of ARDS increased along with CFS score.

Table 6 presents drugs applied during hospitalization in each CFS group. Patients with severe frailty more often needed therapeutic doses of low molecular weight heparin (LMWH) (*p* = 0.011) and other than standard antibiotic therapy (*p* < 0.001), most likely because of more frequent bacterial coinfection. Prednisone was most frequently given in the mild to moderate frailty group (*p* = 0.020). We usually used dexamethasone to decrease immunological response to COVID-19 infection, but we continued with prednisone if it was taken beforehand due to other chronic disease. We also observed significant differences in remdesivir administration: 24.03% of patients in the CFS 1–3 group were treated with remdesivir compared to 3.85% in the CFS 7–9 group (*p* = 0.007).

Table 7 compares registered complications. Frailty was associated with greater risk of cardiological complications (*p* < 0.001) (heart failure (*p* = 0.008), atrial fibrillation (*p* < 0.001) renal complications (*p* < 0.001) (acute kidney injury (AKI) or decompensation of CKD (*p* < 0.001), urinary tract infection (*p* < 0.001)), neurological complications (*p* < 0.001) with impaired consciousness (*p* < 0.001) and other complications (*p* < 0.001) like pressure ulcers (*p* < 0.001), gastrointestinal hemorrhage (*p* = 0.003), or sepsis (*p* < 0.001). Only respiratory failure (*p* = 0.004) and mucosal bleeding (*p* = 0.021) were more frequent in mildly frail patients. Finally, statistical analysis confirmed increased risk of death among frail patients, while most patients (83.72%) with CFS 1–3 were discharged home (*p* < 0.001).

**Figure 1:**
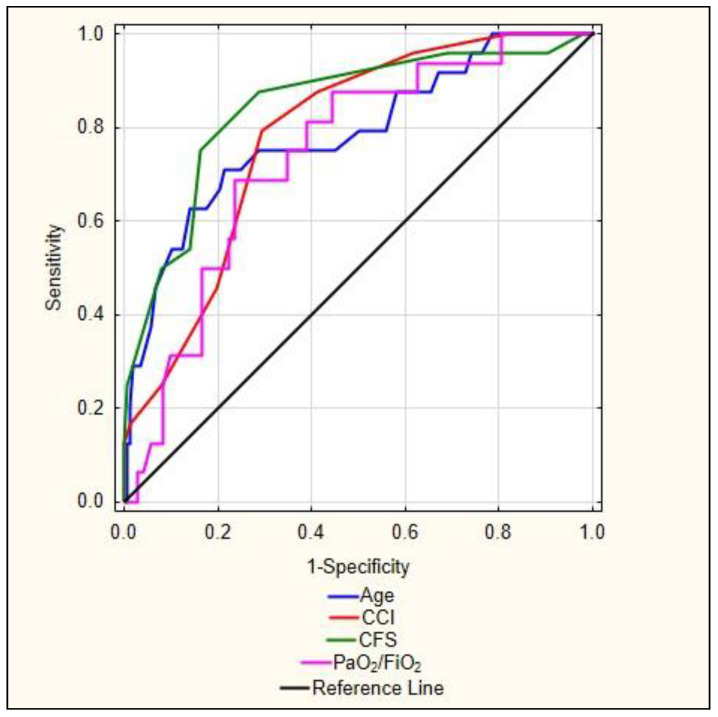
Receiver operating characteristic (ROC) analysis for mortality.

The ROC curves for age, continuous CCI, CFS, and PaO_2_/FiO_2_ are presented in Figure 1. The ROC analysis showed that the CFS is better at predicting the mortality of patients with COVID-19. The data in Table 8 show that CFS has the highest area under the ROC curve (AUC), 0.844, with *p* < 0.001.

**Figure 2:**
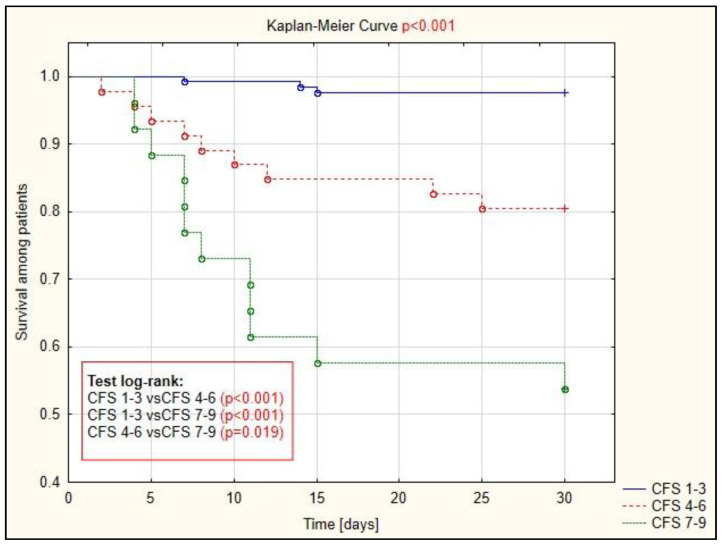
Analysis of survival among patients with COVID-19 with each CFS group.

An assessment of survival probability in each CFS group was performed using the Kaplan–Meier curve (Figure 2). It shows statistically significant differences in 30-day survival between all three groups of patients (*p* < 0.001).

**Figure 3:**
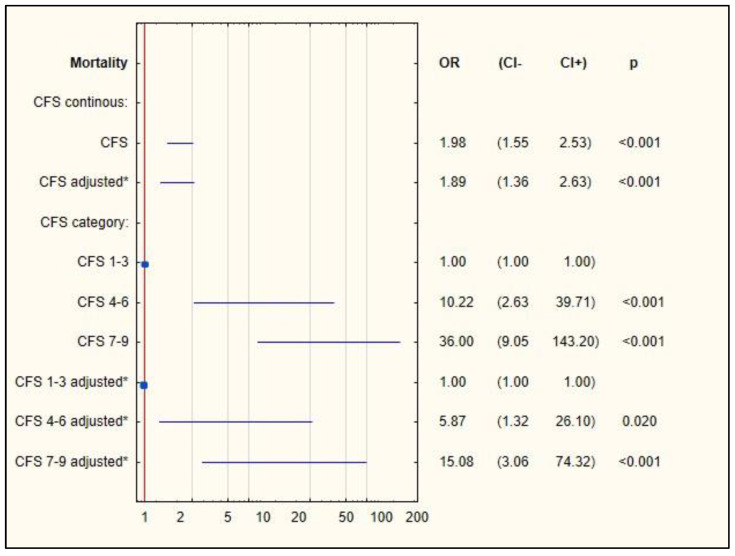
Logistic regression for mortality among patients with COVID-19. Legend: * adjusted by age, gender, BMI, and comorbidities.

A detailed assessment of mortality was performed based on the CFS scale (score 1–9). Logistic regression was performed (Figure 3). The analysis showed a relationship between the occurrence of death and an increase in the CFS score (OR = 1.978, *p* < 0.001). After the results were corrected for data demographics and comorbidities, it was confirmed that mortality was associated with an increase in CFS (OR = 1.89, *p* < 0.001).

## 4. Discussion

Since the emergence of SARS-CoV-2 infection and the declaration of COVID-19 pandemic many patients requiring hospitalization overwhelmed medical systems across the world and therefore it soon became crucial to find a simple tool to identify patients with increased risk of death. The purpose of this study was to evaluate the relationship between frailty on admission to a hospital dedicated to treating patients infected with SARS-CoV-2 with certain outcomes and mortality. The results show that frailty is associated with increased risk of death during hospitalization.

We identified mild and moderate frailty in 22.8% of patients and severe frailty in 13% of patients. Prevalence of frailty in our cohort was lower than reported by other authors, who estimate the prevalence of frailty among hospitalized COVID-19 patients to be between 51% [9] and 54% [10] of patients. 

There may be several reasons why our number of frail patients was lower. First, our ward was a temporary solution to the increased needs of hospitalization of infectious patients, with temporary staff, both nurses and doctors, who work permanently on several other units. Therefore, we hospitalized mostly patients, whose major problem was COVID-19 infection. Those with other severe diseases were often admitted to other hospitals in the area that were also taking care of COVID-19 patients. Second, doctors working in our units do not routinely assess CFS in their clinical practice. A recent study showed that the correlation between CFS scoring by untrained raters and geriatricians may be improved by using a new tool called classification tree. In that study, inexperienced clerks or residents tended to underestimate CFS score compared to geriatricians [11]. Classification trees may guide a rater through the scoring process. This study was published after data collection in our research. Despite the lack of experience and the possibility of underestimating CFS in our study, it still predicted a poor outcome for COVID-19, which proves the utility of this scale. Third, we included all hospitalized patients, not only those aged 65 and more, for whom CSF was created. The use of CFS in younger patients with COVID-19 has already been discussed in several studies [12]. It is possible to underrate young patients suffering from severe diseases or overrate patients with movement disabilities in this group. A high CFS should not be the only admission criterion, but our study showed it is an independent risk factor for death, even when corrected for age, confirming its universality.

Increased risk of complications and death during COVID-19 infection among frail patients has already been reported in several studies. The largest study included 2434 patients from 63 hospitals in 11 countries in Europe [12]. In that study, the risk of in-hospital mortality was significantly higher both in the pre-frail group (defined as CFS 4–5) and the frail group (CFS 6–9) compared the fit group (CFS 1–3) with OR (odds ratio) of 1.54 (95% CI (confidence interval) of 1.16–2.06) and 2.71 (95% CI 2.04–3.6) respectively. In another large analysis of 1376 patients (age 70+) from 15 hospitals in the Netherlands, frailty (CFS 4–5) was associated with a 2-times higher risk of in-hospital mortality (OR 2.0) and severe frailty (CFS 6–9) was associated with almost a 3-times higher risk of death (OR 2.8) [13]. A large meta-analysis of fifteen studies comprising of 23,944 COVID-19 positive patients confirmed that frailty is an independent predictor of mortality among COVID-19 patients [9]. In our study, we also showed a relationship between death and an increase in CFS score (OR 1.98).

The CFS is a tool designed to evaluate physical impairments based on limitations patients experience in their everyday life. It does not consider comorbidities, which may influence survival rates. In our study, frail patients were more likely to have cardiological disorders, diabetes, and CKD, which are known to increase COVID-19 mortality. CCS, HT, and CHF are responsible for approximately a 2-times higher risk of death (RR 2.25 (95% CI 1.6–3.17); RR 1.81 (95% CI 1.43–2.32) and RR 2.03 (95% CI 1.28–3.29) respectively) [14] and the history of CVD is associated with 3-fold increased risk of severe COVID-19 infection and over 11-fold higher risk of all-cause mortality [15]. Mortality among diabetic patients is 2.39-times higher and the risk of severe course of COVID-19 is 1.45-times higher [16]. Mortality among patients with CKD is 3.25 times higher [14]. 

The Charlson Comorbidity Index (CCI) summarizes comorbidities and estimates 10-year survival. The CCI includes several diseases, such as CVD, COPD, diabetes, liver disease, tumors, and others. In our study, frail patients had significantly higher CCI, which is consistent with higher burden of comorbidities found in these groups. The calculated CCI was nearly 6 in severely frail patients, in the moderately frail nearly 5 and in the fit nearly 3 points, which makes an estimated 10-year survival of 2%, 21%, and 77% respectively. The usefulness of CCI in predicting poor outcome of COVID-19 has been evaluated in several studies. The risk of mortality increases by 16% for each increase in CCI [17] and even CCI > 0 is associated with increased risk of severe COVID-19 and death [17,18]. 

We corrected the risk of death for comorbidities, age, sex, and BMI and found that a high CFS score is still significant, independent risk factor of death in patients hospitalized with COVID-19 with OR 1.89 (CI 1.35–2.63, *p* < 0.001)

The negative effect of renin-angiotensin-aldosterone drugs (RAAS) on COVID-19 outcome reported in several studies at the beginning of the pandemic brough attention to a possible relationship between pretreatment with some drug groups and COVID-19 outcome. Therefore, we analyzed what drugs were used by our patients before admission to the hospital (Table 2). Frailer patients had more comorbidities; they used more drugs and were more prone to possible side-effects. Studies on this subject confirmed safety of chronic treatment, which should be continued whenever possible during hospitalization. Large meta-analyses have shown that neither RAAS, nor other antihypertensive drugs, nor NSAIDs are associated with increased risk of death. The reason for previous observations was not the influence of drugs on COVID-19, but it was the effect of underlying comorbidities alone [19,20]. Moreover, statins, due to their anti-inflammatory effect, are associated with lower risk of fatal COVID-19 [21,22,23] among non-ICU patients and lower risk of mechanical ventilation treatment [22]. 

The primary symptoms of COVID-19 are well known and include fever, cough, and dyspnea. On the other hand, there are some atypical manifestations, which occur mainly in elderly, frail patients, such as hypotension, low body temperature, functional decline, and acute mental change [24]. In our study, patients with CFS 1–3 presented mainly olfactory and taste disorders as well as cough. We observed that severely frail patients (CFS 7–9) more frequently complained of gastrointestinal symptoms, such as nausea and vomiting, which may be associated with longer hospitalization and severity of infection [25,26]. This phenomenon can be explained by the viral load and replication within the digestive tract [25]. SARS-CoV2 can invade the human cells by connecting to the angiotensin converting enzyme 2 (ACE-2) receptors, which are localized not only in the alveolar cells in the lung but also in the gastrointestinal tract [26]. Doctors must be aware of atypical symptoms of COVID-19 among older and frail patients. 

The usefulness of some biochemical markers in staging risk in COVID-19 has been studied in many recent analyses. Many investigations focused on LDH and lymphocyte count changes as an independent risk factor of mortality [27,28,29]. Lower lymphocyte counts, higher leukocyte counts, and higher neutrophil-lymphocyte ratio (NLR) were observed in severe cases [28,30,31]. In our study, we did not find a statistically important difference between frailty and LDH level and lymphocyte count, but severely frail patients (CFS 7–9) had higher: WBC, neutrophil count, NLR (neutrophil—leukocyte ratio), D-dimer, and APRI (AST platelet ratio index) and lower HGB and HCT, which is consistent with previous studies [24]. CFS 7–9 was also associated with elevated renal biomarkers (creatinine, urea) and lower GFR.

Patients hospitalized in the Temporary COVID-19 Hospital in Szczecin were treated according to the Polish Society of Epidemiologists and Infectious Disease Doctors guidelines. Treatment strategy includes corticosteroids (patients requiring oxygen therapy due to respiratory failure, most frequently dexamethasone), LMWH (in prophylactic, intermediate, or therapeutic doses, depending on patient’s clinical condition), antibiotics (in case of suspicion of bacterial superinfection; most frequently ceftriaxone, levofloxacin or azithromycin), and remdesivir [32]. Tocilizumab and convalescent plasma were not available in our department. We found that patients with higher frailty stages were treated differently than those less frail. To the best of our knowledge, this is the first analysis that compares COVID-19 treatment depending on CFS score.

Remdesivir is an antiviral agent which can inhibit SARS-CoV-2 replication in vitro [33]. Beigel et al. proved in their randomized, double-blind study that patients who were treated with remdesivir had a shorter recovery and discharge time and had lower mortality in comparison to placebo group [34]. Remdesivir was applied more frequently in the CFS 1–3 group compared to the CFS 7–9 group. The difference in results from elevated biochemical markers of liver and renal dysfunction (alanine aminotransferase > 5-times upper limit of normal and decreased GFR < 30 mL/min) [35] that we observed among frail patients, are contraindications to remdesivir treatment.

The other medications which have proven activity against COVID-19 are glucocorticosteroids, especially dexamethasone. Many recent studies have shown that dexamethasone could reduce mortality [36,37]. In our department, all patients with respiratory failure (defined as SpO_2_ < 94% without oxygen supplementation) received dexamethasone. 

It has been proven that anticoagulant therapy with LMWH is associated with a better prognosis and lower mortality in COVID-19 patients [38]. There are a few mechanisms that lead to prothrombotic state in COVID-19, for example: cytokine storm, pathological complement-activation, endothelial dysfunction caused by hypoxia and virus-related damage which leads to vasoconstriction, platelet activation and aggregation [39]. In our cohort, patients with CFS 7–9 more frequently received LMWH in therapeutic doses due to previously diagnosed atrial fibrillation, deep vein thrombosis, or higher D-dimer levels. We did not observe any differences in occurrence of thromboembolic complications between groups.

We report differences between complications found in patients depending on CFS score. Patients with frailty had higher risk of mucosal bleeding, respiratory failure, and heart failure. Patients with severe frailty suffered from renal, neurological, and infectious complications as well as arrhythmias. These results are similar to those reported by other authors, who mention acute renal failure, delirium and ARDS [40] as the most common. Some of the complications have prognostic significance. Hagg et al. noticed that AKI was an additional risk factor for mortality in the group of patients with CFS > 5 [41]. 

The severity of COVID-19 may be assessed by the intensity of oxygen therapy applied on admission. In our study, patients with CFS 4–6 and CFS 7–9 more often required oxygen supplementation via non-rebreather mask and HFNOT with more intense oxygen flow and oxygen concentration. Those patients had a higher probability of developing severe ARDS. Although this was associated with higher mortality, the qualification and admission rate to ICU was similar in all groups. In a study by Andres-Esteban et al study patients with frailty, defined as CFS ≥ 5 was less often admitted to the ICU than non-frail (CFS 1–3) and pre-frail (CFS = 4) patients [40]. In the COMET study the incidence of ICU admission was higher in the CFS 6–9 group, but lower in CFS 5–6 group compared to patients without frailty. The difference was even more visible in patients younger than 65 years (in the ≥65 years old subgroup there was no statistically significant difference) [12]. Usually, severe frailty may be a contradiction to ICU admission, especially in case of limited resources in the COVID-19 pandemic. In our unit, all patients requiring intensive oxygen therapy were consulted by an ICU-doctor and the decision about admitting them to ICU in case of deterioration was made in advance. Older age, severe comorbidities and severe frailty were the most common reasons for disqualifying patients from ICU treatment.

### Limitations

Our study comes with some limitations. First, the retrospective nature of the study should be noticed. The database was collected from 3 up to 6 months ago. During this time, some changes have been implemented to the general guidelines of COVID-19 treatment (i.e., corticosteroids or antibiotics dosing), thus its potential impact on the final prognosis in particular individuals might remain considerable. Second, we reported only short-term outcomes and did not do any long-term follow-up. Finally, doctors working in our unit were mostly internal medicine and cardiology residents, who do not routinely assess CFS in their clinical practice. As laboratory tests are irrefutable, the approach to assessing CFS may differ among different specialists. Additionally, CFS is only validated for the elderly patients (over 65), and it may not be suitable for assessment of younger people or patients with a learning or progressive disability who were included in the study.

## 5. Conclusions

The presented study provides a comprehensive assessment of frailty in patients infected from SARS COV 2, including respiratory parameters upon admission, treatment, and complications. CFS is an easy scale that may help in the assessment of patients with COVID-19 and in predicting both treatment outcomes and the risk of death. CFS also correlates with other factors influencing the risk of death in COVID-19, such as comorbidities or blood tests results. However, its usefulness should be confirmed in prospective studies, especially in younger patients. 

We think that CFS may be routinely used on admission to a hospital to predict the outcome of treatment. In a pre-hospital setting, it may helpful in deciding whether to admit a patient with COVID-19 to a hospital or not. Among frail patients, even mild symptoms may develop into severe multiorgan disease and result in a patient’s deterioration and death; therefore, it is reasonable to consider hospitalization for all patients with high CFS scores. Those treated at home should be supervised intensively to catch the first moment of deterioration.

## Figures and Tables

**Table 1:** Baseline characteristics of patients included in the study.

Variables	Total (n = 201)	CFS 1–3 (n = 129)	CFS 4–6 (n = 46)	CFS 7–9 (n = 26)	*p*
Demographic data					
Age [years], mean ± SD; Me	68.14 ± 13.82; 69.0	62.76 ± 13.03; 64.0	76.11 ± 9.40; 76.0	80.73 ± 8.34; 82.5	<0.001 *^
Gender [male], n (%)	100 (49.75)	64 (49.61%)	25 (54.35%)	11 (42.31%)	0.636
BMI [kg/m^2^], mean ± SD; Me	27.32 ± 5.21; 27.46	27.49 ± 5; 27.46	27.09 ± 5.37; 27.73	26.87 ± 6.07; 82.5	0.945
Smoking, n (%)	29 (14.43)	24 (19.05)	1 (2.22)	4 (15.38)	0.011
EF [%], (mean ± SD; Me)	47.22 ± 16.11; 55	51.88 ± 17.1; 57.5	45 ± 12.58; 45	40 ± 22.91; 45	0.297
CCI, (mean ± SD; Me)	3.64 ± 2.21; 3	2.70 ± 1.77; 2	4.98 ± 1.72; 5	5.96 ± 2.05; 6	<0.001 *^
Co-morbidities					
Arterial hypertension, n (%)	131 (65.17)	73 (56.59)	34 (73.91)	24 (92.31)	<0.001
Chronic Coronary Syndrome, n (%)	42 (20.9)	17 (13.18)	18 (39.13)	7 (26.92)	<0.001
Myocardial Infarction, n (%)	22 (10.95)	8 (6.2)	11 (23.91)	3 (11.54)	0.005
Chronic Heart Failure, n (%)	36 (17.91)	10 (7.75)	15 (32.61)	11 (42.31)	<0.001
Atrial Fibrillation, n (%)	37 (18.41)	13 (10.08)	15 (32.61)	9 (34.62)	<0.001
Previous Ischemic Stroke, n (%)	15 (7.46)	2 (1.55)	3 (6.52)	10 (38.46)	<0.001
Previous Hemorrhagic Stroke, n (%)	1 (0.49)	0 (0)	0 (0)	1 (3.85)	0.130
Transient Ischemic Attack, n (%)	2 (0.99)	0 (0)	1 (2.17)	1 (3.85)	0.127
CKD (GFR < 60) [mL/min/1.73 m^2^], n (%)	35 (17.41)	14 (10.85)	14 (30.43)	7 (26.92)	0.004
Post-renal Tx, n (%)	4 (1.99)	1 (0.78)	3 (6.52)	0 (0)	0.079
Dialysis, n (%)	4 (1.99)	3 (2.33)	1 (2.17)	0 (0)	0.999
Impaired insulin tolerance, n (%)	5 (2.49)	3 (2.33)	1 (2.17)	1 (3.85)	0.805
Diabetes [oral medications/diet], n (%)	39 (19.4)	21 (16.28)	13 (28.26)	5 (19.23)	0.209
Diabetes [insulin], n (%)	23 (11.44)	5 (3.88)	12 (26.09)	6 (23.08)	<0.001
Gout/hyperuricemia, n (%)	11 (5.47)	5 (3.88)	5 (10.87)	1 (3.85)	0.175
ICA stenosis, n (%)	10 (4.98)	2 (1.55)	4 (8.70)	4 (15.38)	0.004
Chronic peripheral ischemia, n (%)	11 (5.47)	3 (2.33)	3 (6.52)	5 (19.23)	0.003
Previous Venous thrombosis, n (%)	4 (1.99)	0 (0)	1 (2.17)	3 (12.00)	<0.001
Previous Pulmonary Embolism, n (%)	2 (0.99)	1 (0.85)	1 (2.17)	0 (0)	0.627
COPD, n (%)	14 (6.97)	10 (7.75)	3 (6.52)	1 (3.85)	0.920
Asthma, n (%)	16 (7.96)	11 (8.53)	5 (10.87)	0 (0)	0.249
Active neoplasm, n (%)	17 (8.46)	14 (10.85)	2 (4.35)	1 (3.85)	0.265

Legend: CCI—Charlson comorbidity index, CFS—clinical frailty scale, CKD—chronic kidney disease, COPD—chronic obstructive pulmonary disease, CPI—chronic peripheral ischemia, Me—median, BMI—body mass index, EF—ejection fraction, GFR—glomerular filtration rate, ICA—internal carotid artery, n—number of patients, *p*—statistical significance, SD—standard deviation, Tx—transplant. Notes: Statistical significance (*p* < 0.001) in multiple comparison test (* CFS1–3 vs. CFS4–6, ^ CFS1–3 vs. CFS7–9)

**Table 2:** Medications taken by the patients before admission.

Medications	CFS 1–3 (n = 129)	CFS 4–6 (n = 46)	CFS 7–9 (n = 26)	*p*
ASA, n (%)	23 (17.97)	16 (34.78)	10 (38.46)	0.016
ADP inhibitors, n (%)	5 (3.91)	4 (8.89)	2 (7.69)	0.396
OAC/NOAC, n (%)	8 (6.25)	10 (22.22)	6 (23.08)	0.003
B-blockers, n (%)	58 (45.31)	25 (55.56)	17 (65.38)	0.126
ACE-I/Sartans, n (%)	50 (39.06)	22 (48.89)	13 (50.00)	0.375
CCBs, n (%)	22 (17.19)	11 (24.44)	10 (38.46)	0.049
Statins/fibrates, n (%)	24 (18.75)	17 (37.78)	11 (42.31)	0.006
Nitrates, n (%)	0 (0.00)	2 (4.44)	2 (7.69)	0.016
Diuretics, n (%)	39 (30.47)	19 (42.22)	15 (57.69)	0.022
MCRAsm, n (%)	8 (6.25)	8 (17.78)	2 (7.69)	0.066
Bronchodilators, n (%)	19 (14.84)	3 (6.67)	0 (0)	0.050
Oral antidiabetic drugs, n (%)	24 (18.75)	13 (28.89)	4 (15.38)	0.274
Insulin, n (%)	5 (3.91)	12 (26.67)	6 (23.08)	<0.001
Thyroid hormones/thyrostatic, n (%)	21 (16.41)	5 (11.11)	1 (3.85)	0.201
NSAIDs, n (%)	6 (4.69)	1 (2.22)	2 (7.69)	0.559
Immunosuppression, n (%)	5 (3.91)	6 (13.33)	0 (0)	0.025
Opioids, n (%)	2 (1.56)	1 (2.22)	3 (11.54)	0.024

Legend: ASA—acetylsalicylic acid, ADP inhibitors—adenosine diphosphate receptor inhibitors, OAC—oral anticoagulants, NOAC—new oral anticoagulants, ACE-I—angiotensin-converting enzyme inhibitor, CCBs—calcium channel blockers, CFS—clinical frailty scale, MCRAs—mineralocorticoid receptor antagonists, n—number of patients, NSAIDs—nonsteroidal anti-inflammatory drugs, *p*—statistical significance.

**Table 3:** Coronavirus-related symptoms on admission to the hospital.

Symptoms on Admission	CFS 1–3 (n = 129)	CFS 4–6 (n = 46)	CFS 7–9 (n = 26)	*p*
Low-grade fever/Fever, n (%)	86 (66.67)	27 (58.70)	13 (50.00)	0.225
Dyspnea, n (%)	71 (55.04)	28 (60.87)	13 (50.00)	0.641
Cough, n (%)	69 (53.49)	16 (34.78)	7 (26.92)	0.011
Chest pain, n (%)	24 (18.60)	6 (13.04)	3 (11.54)	0.634
Weakness, n (%)	90 (69.77)	33 (71.74)	19 (73.08)	0.947
Nausea, n (%)	19 (14.73)	0 (0)	5 (19.23)	0.003
Vomiting, n (%)	16 (12.40)	0 (0)	4 (15.38)	0.010
Diarrhea, n (%)	18 (13.95)	6 (13.04)	4 (15.38)	0.955
Musculo-articular pains, n (%)	20 (15.50)	5 (10.87)	1 (3.85)	0.284
Lack of taste, n (%)	20 (15.50)	0 (0)	0 (0)	<0.001
Lack of smell, n (%)	15 (11.63)	1 (2.17)	0 (0)	0.042
Headache, n (%)	12 (9.30)	2 (4.35)	0 (0)	0.223

Legend: CFS—clinical frailty scale, n—number of patients, *p*—statistical significance.

**Table 4:** Laboratory results on admission.

	CFS 1–3 (n = 129)	CFS 4–6 (n = 46)	CFS 7–9 (n = 26)	*p*
Laboratory Data on Admission				
HbA_1c_ (%), mean ± SD; Me	6.54 ± 6.05; 1.39	6.91 ± 6.50; 1.55	7.20 ± 6.40; 2.40	0.850
WBC [10^9^/L], mean ± SD; Me	6.65 ± 6.03; 3.33	8.46 ± 6.10; 5.33	9.77 ± 8.41; 5.94	0.011
Neutrophils [10⁹/L], mean ± SD; Me	4.96 ± 4.33; 3.10	6.39 ± 4.24; 4.50	7.91 ± 6.69; 5.36	0.007
Lymphocytes [10^9^/L], mean ± SD; Me	1.14 ± 1.03; 0.71	1.06 ± 1.01; 0.45	1.27 ± 0.91; 1.05	0.929
NLR, mean ± SD; Me	5.85 ± 4.48; 5.28	7.05 ± 5.34; 5.76	8.18 ± 6.62; 5.93	0.044
HGB [mmol/L], mean ± SD; Me	8.08 ± 8.39; 1.36	7.55 ± 7.63; 1.86	7.47 ± 7.65; 1.27	0.008
HCT [l/L], mean ± SD; Me	0.38 ± 0.39; 0.06	0.35 ± 0.35; 0.06	0.36 ± 0.36; 0.06	0.008
Creatinine [mg/dL], mean ± SD; Me	1.10 ± 0.88; 1.08	1.39 ± 1.15; 0.89	2.05 ± 1.44; 2.26	<0.001
GFR [mL/min/1.73 m^2^], mean ± SD; Me	81.80 ± 79.48; 30.20	61.15 ± 58.62; 27.90	58.48 ± 48.71; 43.69	<0.001
Urea [mg/dL], mean ± SD; Me	41.14 ± 37.00; 22.48	62.42 ± 48.90; 38.60	98.63 ± 76.45; 78.24	<0.001
CRP [mg/dL], mean ± SD; Me	69.94 ± 55.69; 57.26	82.09 ± 70.52; 63.84	86.23 ± 54.40; 78.43	0.495
IL-6 [pg/mL], mean ± SD; Me	61.56 ± 37.05; 128.63	63.00 ± 50.05; 65.91	328.16 ± 57.80; 1187.31	0.099
PCT [ng/mL], mean ± SD; Me	0.28 ± 0.11; 0.86	1.01 ± 0.15; 5.03	8.08 ± 0.12; 23.05	0.052
AST [U/L], mean ± SD; Me	46.59 ± 31.00; 59.63	49.60 ± 37.00; 41.42	65.82 ± 37.50; 67.89	0.118
ALT [U/L], mean ± SD; Me	48.19 ± 24.00; 89.85	39.13 ± 26.50; 33.20	36.32 ± 28.00; 30.24	0.987
LDH [U/L], mean ± SD; Me	335.52 ± 318.00; 119.46	369.16 ± 293.00; 180.72	324.25 ± 314.50; 96.13	0.979
Fibrinogen [g/L], mean ± SD; Me	4.91 ± 4.80; 1.78	3.89 ± 3.40; 1.52	4.48 ± 3.20; 2.13	0.166
D-Dimer [ng/mL], mean ± SD; Me	1628.26 ± 804.00; 1848.30	2417.87 ± 1343.00; 2529.88	2917.27 ± 1467.50; 3825.30	0.024
CKMB [U/L], mean ± SD; Me	18.44 ± 17.60; 8.06	27.82 ± 23.00; 16.12	24.47 ± 20.00; 17.83	0.002
TnT [ug/L], mean ± SD; Me	0.02 ± 0.01; 0.02	0.19 ± 0.03; 0.51	0.06 ± 0.03; 0.06	<0.001

Legend: ALT—alanine transaminase, APRI—AST-to-platelet ratio index, APTT—activated partial thromboplastin time, AST—aspartate transaminase, CFS—clinical frailty scale, CK-MB—creatine kinase type MB, CRP—C-reactive protein, GFR—glomerular filtration rate, GGTP—gamma-glutamyl transferase, HbA1C—glycated hemoglobin, HCT—hematocrit, HDL—high-density lipoprotein, HGB—hemoglobin, Il-6—interleukin 6, INR—international normalized ratio, LDH—lactate dehydrogenase, LDL—low-density lipoprotein, Me—median, n—number of patients, NLR—neutrophil-to-lymphocyte ratio, *p*—statistical significance, TC—total cholesterol, TG—triglyceride, PCT—procalcitonin, PLR—platelet-to-lymphocyte ratio, PLT—platelets, PWR—platelet-to-WBC ratio, SD—standard deviation, TnT—troponin T, WBC—white blood cells.

**Table 5:** Respiratory parameters on admission.

		CFS 1–3 (n = 129)	CFS 4–6 (n = 46)	CFS 7–9 (n = 26)	*p*
Respiratory Parameters on Admission					
SpO_2_ (mean ± SD; Me)		94.84 ± 95.00; 2.88	94.54 ± 95.00; 4.03	94.54 ± 96.00; 4.87	0.771
Nasal cannula, n (%)		42 (32.81)	17 (36.96)	7 (26.92)	0.682
Non-rebreather mask, n (%)		13 (10.08)	12 (26.67)	7 (26.92)	0.009
HFNOT + NIV	Yes, n (%)	5 (3.88)	7 (15.22)	3 (11.54)	0.018
	Day started (mean ± SD; Me)	3.80 ± 3.00; 1.92	3.29 ± 3.00; 3.20	5.33 ± 3.00; 5.86	0.694
Flow [L/min] (mean ± SD; Me)		5.71 ± 5.00; 3.71	8.84 ± 6.00; 8.27	9.31 ± 8.00; 5.44	0.009
pH (mean ± SD; Me)		7.47 ± 7.47; 0.06	7.48 ± 7.48; 0.07	7.47 ± 7.49; 0.08	0.437
pO_2_ (mmHg), (mean ± SD; Me)		75.44 ± 71.00; 23.82	70.63 ± 63.00; 21.20	77.59 ± 65.00; 35.88	0.252
pCO_2_ (mmHg), (mean ± SD; Me)		34.51 ± 33.00; 7.49	33.67 ± 34.50; 5.18	32.65 ± 33.00; 5.09	0.816
FiO_2_ (mean ± SD; Me)		0.43 ± 0.23; 0.30	0.57 ± 0.44; 0.28	0.63 ± 0.80; 0.29	0.006 ^
HCO_3_^-^ (mean ± SD; Me)		25.11 ± 25.00; 4.67	25.88 ± 27.00; 5.76	24.65 ± 25.10; 4.90	0.649
BE (mean ± SD; Me)		1.39 ± 1.10; 4.26	3.38 ± 2.70; 5.50	0.37 ± 1.20; 5.57	0.301
PaO_2_/FiO_2_, (mean ± SD; Me)		265.74 ± 285.71; 156.83	181.75 ± 112.50; 155.31	178.92 ± 93.33; 150.76	0.017
ARDS, (n/%)	no	21 (44.68)	4 (16.67)	4 (23.53)	0.034
	mild	9 (19.15)	2 (8.33)	2 (11.76)	
	moderate	6 (12.77)	8 (33.33)	2 (11.76)	
	severe	11 (23.40)	10 (41.67)	9 (52.94)	

Legend: ARDS—acute respiratory distress syndrome, BE—base excess, CFS—clinical frailty scale, FiO_2_—fraction of inspired oxygen, HFNOT—high-flow nasal oxygen therapy, Me—median, n—number of patients, NIV—non-invasive ventilation, *p*—statistical significance, pCO_2_—partial pressure of carbon dioxide, PaO_2_—partial pressure of oxygen, SD—standard deviation, SpO_2_—peripheral oxygen saturation, PaO_2_/FiO_2_-oxygenation index. Notes: Statistical significance (*p* < 0.001) in multiple comparison test (^ CFS1–3 vs. CFS7–9).

**Table 6:** Data regarding COVID-19 specific treatment during hospitalization.

	CFS 1–3 (n = 129)	CFS 4–6 (n = 46)	CFS 7–9 (n = 26)	*p*
COVID-19 Specific Treatment				
LMWH, n (%)	123 (95.35)	44 (95.65)	24 (92.31)	0.791
Prophylactic dose [40 mg once a day], n (%)	52 (40.31)	23 (50.00)	4 (15.38)	0.012
Intermediate dose [1 mg/kg once a day], n (%)	50 (38.76)	14 (30.43)	9 (34.62)	0.612
Therapeutic dose [1 mg/kg twice a day], n (%)	29 (22.48)	16 (34.78)	13 (50.00)	0.011
Antibiotic therapy, n (%)	117 (90.70)	40 (86.96)	25 (96.15)	0.436
Ceftriaxone, n (%)	103 (79.84)	35 (76.09)	24 (92.31)	0.234
Azithromycin, n (%)	86 (66.67)	34 (73.91)	22 (84.62)	0.169
Levofloxacin, n (%)	11 (8.53)	4 (8.70)	2 (7.69)	0.998
Another antibiotic, n (%)	16 (12.40)	7 (15.22)	15 (57.69)	<0.001
Steroid therapy, n (%)	90 (69.77)	36 (78.26)	19 (73.08)	0.542
Dexamethasone, n (%)	89 (68.99)	30 (65.22)	19 (73.08)	0.784
Prednisone, n (%)	1 (0.78)	4 (8.70)	0 (0.00)	0.020
Hydrocortisone, n (%)	2 (1.55)	3 (6.52)	2 (7.69)	0.133
Another steroid, n (%)	0 (0)	1 (2.17)	1 (3.85)	0.127
Max. dexamethasone dose (or equivalent) (mean ± SD; Me)	6.07 ± 4.00; 2.76	12.19 ± 8.00; 32.38	7.58 ± 8.00; 3.81	0.140
Time of steroid therapy [days] (mean ± SD; Me)	8.49 ± 7.00; 5.45	8.39 ± 8.50; 5.12	5.89 ± 4.00; 4.85	0.059
Vitamin D3, n (%)	35 (27.13)	11 (23.91)	6 (23.08)	0.915
Remdesivir, n (%)	31 (24.03)	4 (8.70)	1 (3.85)	0.007

Legend: CFS—clinical frailty scale, LMWH—low molecular weight heparin, Me—median, n—number of patients, *p*—statistical significance, SD—standard deviation.

**Table 7:** Complications and follow-up in COVID-19 patients.

		CFS 1–3 (n = 129)	CFS 4–6 (n = 46)	CFS 7–9 (n = 26)	*p*
Complications					
Cardiological complications (n/%)		4 (3.10)	11 (23.91)	9 (34.62)	<0.001
Heart Failure, n (%)		2 (1.55)	5 (10.87)	3 (11.54)	0.008
Myocardial Infarction, n (%)		0 (0)	1 (2.17)	0 (0)	0.358
Atrial Fibrillation, n (%)		3 (2.33)	5 (10.87)	6 (23.08)	<0.001
Atrial Flutter, n (%)		0 (0)	0 (0)	1 (3.85)	0.129
Other arrhythmias (including ventricular, supraventricular arrhythmias and atrioventricular conduction disorders), n (%)		1 (0.78)	1 (2.17)	0 (0)	0.589
Pulmonary complications, n (%)		89 (68.99)	32 (69.57)	19 (73.08)	0.948
Respiratory failure (pO_2_ < 60 mmHg and/or pCO_2_ > 45 mmHg), n (%)		20 (15.50)	17 (36.96)	9 (34.62)	0.004
Radiological signs of pneumonia, n (%)		114 (88.37)	38 (84.44)	21 (80.77)	0.454
Clinical manifestation of pneumonia, n (%)		95 (73.64)	36 (78.26)	21 (80.77)	0.758
Fibrosis, n (%)		5 (3.88)	2 (4.44)	2 (7.69)	0.605
Pneumothorax, n (%)		2 (1.55)	1 (2.22)	0 (0)	0.999
Hydrothorax, n (%)		10 (7.75)	9 (20.00)	3 (11.54)	0.074
Renal complications, n (%)		9 (6.98)	12 (26.09)	14 (53.85)	<0.001
AKI or decompensation of CKD (creatinine level ratio (last measurement/admission)), n (%)		8 (6.20)	13 (28.26)	12 (46.15)	<0.001
Urinary Tract Infection, n (%)		5 (3.88)	6 (13.04)	8 (30.77)	<0.001
Neurological complications, n (%)		8 (6.20)	9 (19.57)	13 (50.00)	<0.001
Impaired consciousness, n (%)		10 (7.75)	14 (30.43)	15 (57.69)	<0.001
Transient Ischemic Attack, n (%)		0 (0)	1 (2.17)	0 (0)	0.358
Ischemic stroke, n (%)		1 (0.78)	0 (0)	0 (0)	0.999
Seizures, n (%)		1 (0.78)	0 (0)	0 (0)	0.999
Venous thromboembolism (n/%)		3 (2.33)	2 (4.35)	1 (3.85)	0.561
Venous thrombosis, n (%)		1 (0.78)	0 (0)	0 (0)	0.999
Pulmonary Embolism, n (%)		3 (2.33)	2 (4.35)	1 (3.85)	0.561
Other complications, n (%)		5 (3.88)	5 (10.87)	10 (38.46)	<0.001
Pressure ulcers, n (%)		1 (0.78)	0 (0)	4 (15.38)	<0.001
Gastrointestinal hemorrhage, n (%)		0 (0)	2 (4.35)	3 (11.54)	0.003
Mucosal bleeding, n (%)		0 (0)	3 (6.52)	1 (3.85)	0.021
HIT, (n/%)		0 (0)	1 (2.17)	0 (0)	0.358
Sepsis, n (%)		7 (5.43)	0 (0)	8 (30.77)	<0.001
C. difficile, (n/%)		2 (1.55)	1 (2.17)	1 (3.85)	0.589
FOLLOW-UP					
Time of stay in ward (including the day of admission and discharge) (mean ± SD; Me)		10.43 ± 9.00; 6.07	11.00 ± 11.00; 6.43	11.27 ± 10.50; 6.21	0.724
Discharge, (n/%)	home	108 (83.72)	31 (67.39)	11 (42.31)	<0.001
	another department	10 (7.75)	2 (4.35)	1 (3.85)	
	ICU	8 (6.20)	4 (8.70)	2 (7.69)	
	death	3 (2.33)	9 (19.57)	12 (46.15)	

Legend: AKI—acute kidney injury, CFS—clinical frailty scale, CKD—chronic kidney disease, HIT—heparin-induced thrombocytopenia, ICU—intensive care unit, n—number of patients, OR—odds ratio, *p*—statistical significance.

**Table 8:** Receiver operating characteristic (ROC) analysis for mortality.

Mortality.				
Variable	AUC	AUC − 95%	AUC + 95%	*p*-Value
**Age**	0.784	0.678	0.891	<0.001
**CCI**	0.783	0.700	0.865	<0.001
**CFS**	0.844	0.754	0.935	<0.001
**PaO_2_/FiO_2_**	0.741	0.618	0.865	<0.001

Legend: AUC—area under the curve, PaO_2_/FiO_2_-oxygenation index

## Data Availability

Data will be available upon request.

## References

[1] Galloway J.B., Norton S., Barker R.D., Brookes A., Carey I., Clarke B.D., Jina R., Reid C., Russell M.D., Sneep R. (2020). A clinical risk score to identify patients with COVID-19 at high risk of critical care admission or death: An observational cohort study. J. Infect..

[2] Booth A., Reed A.B., Ponzo S., Yassaee A., Aral M., Plans D., Labrique A., Mohan D. (2021). Population risk factors for severe disease and mortality in COVID-19: A global systematic review and meta-analysis. PLoS ONE.

[3] Rockwood K., Song X.W., MacKnight C., Bergman H., Hogan D.B., McDowell I., Mitnitski A. (2005). A global clinical measure of fitness and frailty in elderly people. CMAJ.

[4] Pulok M.H., Theou O., van der Valk A., Rockwood K. (2020). The role of illness acuity on the association between frailty and mortality in emergency department patients referred to internal medicine. Age Ageing.

[5] NICE (2021). COVID-19 Rapid Guideline: Managing COVID-19.

[6] Kotfis K., Witkiewicz W., Szylińska A., Witkiewicz K., Nalewajska M., Feret W., Wojczyński Ł., Duda Ł., Ely E. (2021). Delirium Severely Worsens Outcome in Patients with COVID-19—A Retrospective Cohort Study from Temporary Critical Care Hospitals. J. Clin. Med..

[7] Rockwood K., Theou O. (2020). Using the Clinical Frailty Scale in Allocating Scarce Health Care Resources. Can. Geriatr. J..

[8] Pranata R., Henrina J., Lim M.A., Lawrensia S., Yonas E., Vania R., Huang I., Lukito A.A., Suastika K., Kuswardhani R.T. (2020). Clinical frailty scale and mortality in COVID-19: A systematic review and dose-response meta-analysis. Arch. Gerontol. Geriatr..

[9] Zhang X.-M., Jiao J., Cao J., Huo X.-P., Zhu C., Wu X.-J., Xie X.-H. (2021). Frailty as a predictor of mortality among patients with COVID-19: A systematic review and meta-analysis. BMC Geriatr..

[10] Verholt A.B., Gregersen M., Gonzalez-Bofill N., Hansen T.K., Ebdrup L., Foss C.H., Lietzen L.W. (2021). Clinical presentation and outcomes of COVID-19 in older hospitalised patients assessed by the record-based multidimensional prognostic index, a cross-sectional study. Eur. Geriatr. Med..

[11] Theou O., Pérez-Zepeda M.U., van der Valk A.M., Searle S.D., Howlett S.E., Rockwood K. (2021). A classification tree to assist with routine scoring of the Clinical Frailty Scale. Age Ageing.

[12] Sablerolles R.S.G., Lafeber M., van Kempen J.A.L., van de Loo B.P.A., Boersma E., Rietdijk W.J.R., Polinder-Bos H.A., Mooijaart S.P., van der Kuy H., Versmissen J. (2021). Association between Clinical Frailty Scale score and hospital mortality in adult patients with COVID-19 (COMET): An international, multicentre, retrospective, observational cohort study. Lancet Heal. Longev..

[13] Blomaard L.C., van der Linden C.M.J., van der Bol J.M., Jansen S.W.M., Polinder-Bos H.A., Willems H.C., Festen J., Barten D.G., Borgers A.J., Bos J.C. (2021). Frailty is associated with in-hospital mortality in older hospitalised COVID-19 patients in the Netherlands: The COVID-OLD study. Age Ageing.

[14] Ssentongo P., Ssentongo A.E., Heilbrunn E.S., Ba D.M., Chinchilli V.M. (2020). Association of cardiovascular disease and 10 other pre-existing comorbidities with COVID-19 mortality: A systematic review and meta-analysis. PLoS ONE.

[15] Aggarwal G., Cheruiyot I., Aggarwal S., Wong J., Lippi G., Lavie C.J., Henry B.M., Sanchis-Gomar F. (2020). Association of Cardiovascular Disease with Coronavirus Disease 2019 (COVID-19) Severity: A Meta-Analysis. Curr. Probl. Cardiol..

[16] Kaminska H., Szarpak L., Kosior D., Wieczorek W., Szarpak A., Al-Jeabory M., Gawel W., Gasecka A., Jaguszewski M.J., Jarosz-Chobot P. (2021). Impact of diabetes mellitus on in-hospital mortality in adult patients with COVID-19: A systematic review and meta-analysis. Acta Diabetol..

[17] Tuty Kuswardhani R.A., Henrina J., Pranata R., Anthonius Lim M., Lawrensia S., Suastika K. (2020). Charlson comorbidity index and a composite of poor outcomes in COVID-19 patients: A systematic review and meta-analysis. Diabetes Metab. Syndr. Clin. Res. Rev..

[18] Christensen D.M., Strange J.E., Gislason G., Torp-Pedersen C., Gerds T., Fosbøl E., Phelps M.D. (2020). Charlson Comorbidity Index Score and Risk of Severe Outcome and Death in Danish COVID-19 Patients. J. Gen. Intern. Med..

[19] Bezabih Y.M., Bezabih A., Alamneh E., Peterson G.M., Bezabhe W. (2021). Comparison of renin–angiotensin–aldosterone system inhibitors with other antihypertensives in association with coronavirus disease-19 clinical outcomes. BMC Infect. Dis..

[20] Kow C.S., Hasan S.S. (2021). The risk of mortality in patients with COVID-19 with pre-diagnosis use of NSAIDs: A meta-analysis. Inflammopharmacology.

[21] Chow R., Im J., Chiu N., Chiu L., Aggarwal R., Lee J., Choi Y.-G., Prsic E.H., Shin H.J. (2021). The protective association between statins use and adverse outcomes among COVID-19 patients: A systematic review and meta-analysis. PLoS ONE.

[22] Wu K.-S., Lin P.-C., Chen Y.-S., Pan T.-C., Tang P.-L. (2021). The use of statins was associated with reduced COVID-19 mortality: A systematic review and meta-analysis. Ann. Med..

[23] Onorato D., Pucci M., Carpene G., Henry B.M., Sanchis-Gomar F., Lippi G. (2021). Protective Effects of Statins Administration in European and North American Patients Infected with COVID-19: A Meta-Analysis. Semin. Thromb. Hemost..

[24] Poco P.C.E., Aliberti M.J.R., Dias M.B., Takahashi S.D.F., Leonel F.C., Altona M., Venys A.L., Shin-Ike I.A., Garcia B.A., Sumita L.H. (2020). Divergent: Age, Frailty, and Atypical Presentations of COVID-19 in Hospitalized Patients. J. Gerontol. Ser. A.

[25] Pan L., Mu M., Yang P., Sun Y., Wang R., Yan J., Li P., Hu B., Wang J., Hu C. (2020). Clinical Characteristics of COVID-19 Patients with Digestive Symptoms in Hubei, China: A Descriptive, Cross-Sectional, Multicenter Study. Am. J. Gastroenterol..

[26] Garland V., Kumar A.B., Borum M.L. (2020). Gastrointestinal and Hepatic Manifestations of COVID-19: Evolving Recognition and Need for Increased Understanding in Vulnerable Populations. J. Natl. Med. Assoc..

[27] Henry B.M., Aggarwal G., Wong J., Benoit S., Vikse J., Plebani M., Lippi G. (2020). Lactate dehydrogenase levels predict coronavirus disease 2019 (COVID-19) severity and mortality: A pooled analysis. Am. J. Emerg. Med..

[28] Qin C., Zhou L., Hu Z., Zhang S., Yang S., Tao Y., Xie C., Ma K., Shang K., Wang W. (2020). Dysregulation of Immune Response in Patients with Coronavirus 2019 (COVID-19) in Wuhan, China. Clin. Infect. Dis..

[29] Bartziokas K., Kostikas K. (2020). Lactate dehydrogenase, COVID-19 and mortality. Med. Clin..

[30] De Smet R., Mellaerts B., Vandewinckele H., Lybeert P., Frans E., Ombelet S., Lemahieu W., Symons R., Ho E., Frans J. (2020). Frailty and Mortality in Hospitalized Older Adults With COVID-19: Retrospective Observational Study. J. Am. Med. Dir. Assoc..

[31] Jimeno S., Ventura P.S., Castellano J.M., García-Adasme S.I., Miranda M., Touza P., Lllana I., López-Escobar A. (2020). Prognostic implications of neutrophil-lymphocyte ratio in COVID-19. Eur. J. Clin. Investig..

[32] Flisiak R., Parczewski M., Horban A., Jaroszewicz J., Kozielewicz D., Pawłowska M., Piekarska A., Simon K., Tomasiewicz K., Zarębska-Michaluk D. (2020). Management of SARS-CoV-2 infection: Recommendations of the Polish Association of Epidemiologists and Infectiologists. Annex no. 2 as of October 13, 2020. Pol. Arch. Intern. Med..

[33] Sheahan T.P., Sims A.C., Leist S.R., Schäfer A., Won J., Brown A.J., Montgomery S.A., Hogg A., Babusis D., Clarke M.O. (2020). Comparative therapeutic efficacy of remdesivir and combination lopinavir, ritonavir, and interferon beta against MERS-CoV. Nat. Commun..

[34] Beigel J.H., Tomashek K.M., Dodd L.E., Mehta A.K., Zingman B.S., Kalil A.C., Hohmann E., Chu H.Y., Luetkemeyer A., Kline S. (2020). Remdesivir for the Treatment of COVID-19—preliminary report. N. Engl. J. Med..

[35] Singh A.K., Singh A., Singh R., Misra A. (2020). Remdesivir in COVID-19: A critical review of pharmacology, pre-clinical and clinical studies. Diabetes Metab. Syndr. Clin. Res. Rev..

[36] Ahmed M.H., Hassan A. (2020). Dexamethasone for the Treatment of Coronavirus Disease (COVID-19): A Review. SN Compr. Clin. Med..

[37] The RECOVERY Collaborative Group (2021). Dexamethasone in Hospitalized Patients with Covid-19. N. Engl. J. Med..

[38] Tang N., Bai H., Chen X., Gong J., Li D., Sun Z. (2020). Anticoagulant treatment is associated with decreased mortality in severe coronavirus disease 2019 patients with coagulopathy. J. Thromb. Haemost..

[39] Carfora V., Spiniello G., Ricciolino R., Di Mauro M., Migliaccio M.G., Mottola F.F., Verde N., Coppola N., Vanvitelli COVID-19 Group (2020). Anticoagulant treatment in COVID-19: A narrative review. J. Thromb. Thrombolysis.

[40] Andrés-Esteban E.M., Quintana-Diaz M., Ramírez-Cervantes K.L., Benayas-Peña I., Silva-Obregón A., Magallón-Botaya R., Santolalla-Arnedo I., Juárez-Vela R., Gea-Caballero V. (2021). Outcomes of hospitalized patients with COVID-19 according to level of frailty. PeerJ..

[41] Hägg S., Jylhävä J., Wang Y., Xu H., Metzner C., Annetorp M., Garcia-Ptacek S., Khedri M., Boström A.-M., Kadir A. (2020). Age, Frailty, and Comorbidity as Prognostic Factors for Short-Term Outcomes in Patients with Coronavirus Disease 2019 in Geriatric Care. J. Am. Med. Dir. Assoc..

